# Continuous Use of Combined Hormonal Contraceptive and the Effect on Blood Coagulation Factors in Female Capuchin Monkeys (*Sapajus libidinosus*)

**DOI:** 10.1155/2019/2047803

**Published:** 2019-03-13

**Authors:** Rosângela C. Rodrigues, Flávia Schechtman Belham, Ana Garcia, Corina Satler, Carlos Tomaz, Maria Clotilde H. Tavares

**Affiliations:** ^1^Department of Biological Sciences, State University of Feira de Santana, Avenida Transnordestina, s/n - Novo Horizonte CEP, 44036-900 Feira de Santana, Bahia, Brazil; ^2^Laboratory of Neuroscience and Behaviour, University of Brasilia, Brasilia, Brazil; ^3^Institute of Cognitive Neuroscience, University College London (UCL), London WC1N 3AZ, UK; ^4^Euro-American University Center (UNIEURO), Brasilia, Brazil; ^5^Faculty of Ceilandia, University of Brasilia, Brasilia, Brazil; ^6^Neuroscience Research Program, CEUMA University, São Luís, Brazil

## Abstract

This study aimed at evaluating the availability of the primate *Sapajus libidinosus* as an animal model for research assessing the physiological effects of the continuous use of combined hormonal contraceptives. In order to do this, six reproductively active female *S. libidinosus* from the Primate Research Center of the University of Brasília were selected to take part in this experiment. Every 21 days or so, each female received a single dose of combined hormonal contraceptive (algestone acetophenide and 17-enanthate estradiol) in a total of five doses throughout the experiment. The physiological parameters were accessed by means of 13 blood samples from each female, whereas three were gathered during the baseline and 10 samples were collected during the treatment phase. The results showed that the contraceptive use provoked changes in hematological coagulation factors such as an increase in the amount of platelets (*p* = 0.039) and a reduction in both prothrombin (*p* < 0.001) and thromboplastin coagulation time (*p* < 0.001). These results are similar to what has been observed in human patients; thus, it is concluded that *S. libidinosus* can be successfully used in studies about the physiological impact of hormonal contraceptives.

## 1. Introduction

Hormonal contraceptives are synthetic hormones composed of solely progestogen or of a combination of estrogen and progestogen. These synthetic hormones are similar to those produced by the ovaries, and the inhibition of ovulation caused by the contraceptive is a result of the inhibition of both follicle-stimulating hormone (FSH) and luteinizing hormone (LH) [1]. The use of hormonal contraceptives, available since 1960, is the most popular and efficient pregnancy-preventing method. These contraceptives are also widely used for the treatment of clinical conditions such as endometriosis, polycystic ovary, premenstrual syndrome, heavy menstrual bleeding, and menstrual cramps [2, 3]. The benefits of the use of hormonal contraceptives are numerous and unquestionable. Among others, it has allowed female emancipation and the insertion of women in the workforce, besides the improvements in birth control and family planning [4, 5]. Since its invention, over 100 million women all over the world have used synthetic hormones to suppress ovulation [6, 7].

However, despite the benefits, some side effects of the contraceptives can pose a serious threat to the users' health [8]. For example, an increase in susceptibility has been noticed for strokes (AVC) [8, 9], heart disease [10], pulmonary embolism [11], and breast tumors [12]. The side effects of contraceptives are especially concerning when considering their effects on blood coagulation factors, since many studies directly associate the use of hormonal contraceptives with an increase in thrombosis risk [8–10]. Thus, it is of paramount importance to conduct studies that focus on these side effects and in how hormonal contraceptives affect female physiological integrity.

The present study aimed at evaluating the effects of the chronic use of a combined hormonal contraceptive (CHC) containing 150 mg of algestone acetophenide (dihydroxyprogesterone) and 10 mg of 17-enanthate estradiol on physiological aspects of female capuchin monkeys (*Sapajus libidinosus*). This research also aimed at verifying the viability of this species as animal models in studies related to the use of hormonal contraceptives directed for human use. These primates were chosen because females of this species have an ovarian-type menstrual cycle that lasts around 20 days [13, 14] and, just as in women, estrogens also act during the follicular phase and progestogens in the luteal phase [14].

## 2. Materials and Methods

### 2.1. Ethical Aspects

The present study was approved by the ethics committee on Animal Research of the Medical School of the University of Brasília, according to the document UnBdoc 101375/2011. All of the ethical precepts associated with animal research established by the Brazilian School of Animal Experimentation (COBEA) were rigorously met and executed.

### 2.2. Animals

For this study, six female capuchin monkeys (*Sapajus libidinosus*) with ages between six and 15 were used. These individuals were kept in groups composed of two females and one adult male. The males were only used as social companions to the females. It should be noted that all females underwent the same experimental conditions, that is, the same social, nutritional, and environmental conditions. The animals used in this experiment were kept at the Primate Center of the University of Brasília (CP-UnB), registered under the number 1/53/1999/000006-2 and according to the laws on keeping native animals in captivity determined by the local environmental agency, the Brazilian Institute of the Environment (IBAMA).

The enclosures where the animals are kept at the CP-UnB are immersed in a gallery forest subject to natural light, temperature, and humidity variations. Each enclosure measures 4 m × 2 m × 3 m (depth, width, and height). Inside, there is a nesting box that works as a shelter for the animals. There are also ropes and perches for environmental enrichment purposes. The animals' diet consists of fresh fruits and vegetables once a day and water *ad libitum*. The overall keeping routine was not altered for this study, and throughout the experiment, the animals were clinically evaluated by the veterinarian staff at the CP-UnB.

### 2.3. Combined Hormonal Contraceptive (CHC) Administration

The chosen CHC for this experiment is a combination of estrogen and progesterone (Perlutan®, Boehringer Ingelheim), in which each 1 ml ampoule is composed of 150 mg of algestone acetophenide (dihydroxyprogesterone) and 10 mg of 17-enanthate estradiol. The proper individual dosage was calculated before the CHC administration by the allometric formula BMR = *K*^∗^*M*^0.75^ [15]. BMR corresponds to the basal metabolic rate, *M* is the individual's weight in kilograms, and *K* is a taxonomically dependent constant, based on core body temperature. The hormonal contraceptive was administered at the end of the 21-day menstrual cycle described for this species [13].

### 2.4. Blood Sampling and Physiological Analyses

The subjects were individually captured by the keepers with the aid of large nets and taken to the biomedical procedure room of the CP-UnB. All animals were anesthetized with isoflurane and 100% oxygen through a portable anesthetic machine (Vetcase®, Brasmed, Brazil). Physiological parameters (temperature, heartbeat, and respiratory frequency) were checked in order to monitor the anesthetic procedure. When the animals showed signs of sedation, 8-10 ml of blood were collected from the femoral vein. The order in which the females were submitted to this procedure was kept the same throughout the whole experiment. The blood samples were taken in the morning between 8:00 am and 12:00 pm. The time spent with the capture, the blood sampling, and the full recovery of the animals varied between five and 30 minutes, depending on the individual.

Part of the blood was stored in evacuated blood tubes (Vacuette®, Brazil) without anticoagulants for serological sex hormones (estradiol, estrone, testosterone, and dihydrotestosterone (DHT)) and cortisol, human chorionic gonadotropin, hemoglobin, sex-hormone-binding globulin, and lipids. Another part was stored in tubes with EDTA anticoagulant and used to evaluate the number of platelets. The remaining was stored in tubes with citrate for blood coagulation factor analyses such as fibrinogen, prothrombin, and thromboplastin time. The tubes were labeled and stored in a container with dry ice until transportation to SABIN Laboratory in the same city as the CP-UnB, where the hormonal analyses were made. At the laboratory, they were centrifuged at 3000 rpm for 5 min at room temperature, and the supernatant (serum or plasma) was collected and frozen at -20°C.

The serum was analyzed according to its concentration of physiological parameters (diluted to a factor of 1 : 50; L2M1Z diluent kits, Diagnostic Products Corp., Los Angeles, CA, USA) using commercial kits provided by Siemens Medical Solutions USA, Inc. for assay chemiluminescence enzyme immunoassay IMMULITE® automated system. Intra- and interassay coefficients of variation were 9.4% and 7.4%, respectively, for the hormonal parameters (estradiol, estrone, testosterone, and dihydrotestosterone (DHT)) and cortisol.

The sensitivity of hormones was 0.20 ng/dl for cortisol (LKCO1 IMMULITE® 2000), 15 ng/dl for total testosterone (LSUBX IMMULITE® 2000), 15 pg/ml for estradiol (LKE21 IMMULITE® 1000), 0.3 ng/ml for DHT (LKAO1 IMMULITE® 1000) and human chorionic gonadotropin (LKFS1 IMMULITE®2000), and 0.268 ng/ml for SHBG (LKSH1 IMMULITE® 1000).

From each blood sample obtained, platelets, erythrocytes, and leukocytes were counted with the manual method in the chamber of Neubauer. The concentration of platelets, erythrocytes, and leukocytes was determined by the percentage of variables in relation to whole blood. The concentration of the TGF-*β*1 growth was measured by commercial ELISA kit sandwich with an absorbance of 450 nm used in the quantification of TGF-*β*1 in cell culture, plasma and human serum, rat, and bovine. A Multiskan 10 device was used in the incubation steps with plate agitation and reading in nm. The coefficient curve correlation was 0.9996, and the CV (%) intra- and interassay were 27.86 and 27.77, respectively.

Factors associated with blood coagulation were analyzed using an automated coagulometer method for the determination of the prothrombin time in citrated plasma. For this, we used TP BIOCLIN K089 kits with prothrombin time (quick) of 10 to 14 seconds and activity of 70 to 100%. This method consists of the measurement of the plasma coagulation time after the addition of a source of thromboplastin (factor III, tissue factor) and calcium. Factor VII is then activated, forming a complex (FT-FVIIa complex) that will activate factors IX and X. Factor Xa along with the tissue factor phospholipids, factor Va, and calcium form the prothrombin activator complex that transforms the prothrombin (factor II) in thrombin (factor IIa). Thrombin acts on fibrinogen by turning it into fibrin. This test the factors of the prothrombin complex (factors II, V, VII, and X).

The determination of the partial thromboplastin time in citrated plasma was done with a method that consists of determining the coagulation time of citrated plasma, after the addition of reagents containing a plasma activator (Elágico) and phospholipids, which will act as substitutes for platelets. Initially, the plasma was incubated with the activator at 37° for 3 minutes. Then, the clotting agent calcium was added for recalcification, triggering the coagulation mechanism of the intrinsic pathway. This stage must be timed until the formation of the clot.

The quantitative determination of fibrinogen was done with the method of Biocontrol Coagulation N and Biocontrol Coagulation P kits BIOCLIN. This method consists of the quantitative determination of fibrinogen-citrated plasma, after the addition of a standardized quantity of thrombin in a plasma sample-diluted citrate and coagulation time measurement. When the thrombin concentration is high, the coagulation of the diluted citrated plasma is inversely proportional to fibrinogen concentration. To determine the concentration of fibrinogen, coagulation time of the analyzed plasma is compared with the coagulation of another plasma sample with known fibrinogen concentration.

The lipid profile was evaluated by means of total cholesterol, LDL-cholesterol, HDL-cholesterol, and triglycerides, according to reference values Kwiterovich 12. This was done with the method of colorimetric enzymatic, using LABTEST reagents. The LDL-cholesterol values were obtained by means of the Friedewald equation. The concentration of lipid oxidation products in plasma (MDA) was obtained after determining the reaction product between the thiobarbituric acid (TBA) and the aldehydes produced during lipid oxidation.

The concentration of hemoglobin was calculated in the field based on the cyanmethemoglobin method. The method is based on the oxidation of the iron (iron II) atom of the molecule of hemoglobin by potassium ferricyanide at weakly alkaline pH, forming methemoglobin which is converted to cyanmethemoglobin after the reaction with the potassium cyanide. The reddish color is proportional to the hemoglobin concentration present in the sample. This is a reliable method to determine the concentration of hemoglobin in the field. In the case of hemoglobin measurement of less than 9 g/dl, a second analysis was performed and the mean between the two measurements was adopted as final.

### 2.5. Study Design

The study lasted 126 days. This period was divided into a baseline (BL) followed by five consecutive hormonal contraceptive treatments (CHC). The BL lasted for 21 days (one menstrual cycle), during which three blood samples were taken from each individual. The first sampling was made on the first day of the experiment, the second on the 10^th^ day and the third on the 21^st^ day. It should be noted that females did not receive contraceptive hormones during the BL phase.

**Figure 1:**
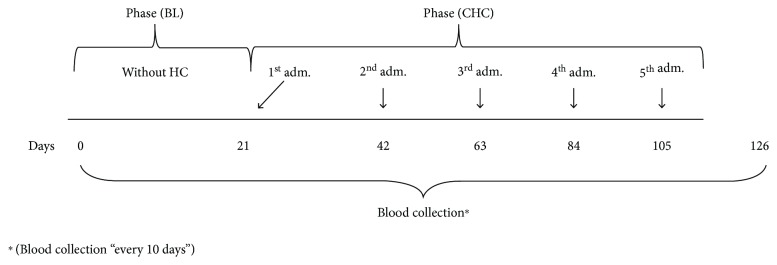
Experimental design where blood sampling is represented as well as the administration of hormonal contraceptive during the BL (baseline) and CHC (combined hormonal contraceptive) phases in six female capuchin monkeys.

The hormonal contraceptive phase lasted for the remaining 105 days of the experiment (five consecutive menstrual cycles), during which each female received a total of five doses of CHC. The first contraceptive administration took place on the 21^st^ day of the experiment, that is, the last BL day. The following doses were administered every 21 days. For the physiological parameter analyses, 10 blood samples were collected per female in a fixed ±10-day interval (Figure 1). This procedure was rigorously followed in order to guarantee the samples from both menstrual phases, one corresponding to the follicular phase (midcycle) and one respective to the luteal phase (end of cycle).

### 2.6. Statistical Analyses

The data was analyzed using the statistical software Statistical Analysis System (SAS®, Cary, North Carolina, v.9.3) independently for each physiological parameter.

First, the behavior of the variables was checked for consistency, and the frequency distribution was observed using the Kolmogorov-Smirnov test. Initially, the three BL samples were averaged and compared to the average of the 10 CHC samples by applying the multivariate analyses of variance (MANOVA). If this difference was significant, an average test (Tukey) was conducted between the BL average and the CHC samples collected in each menstrual cycle. The aim of this study was to analyze the long-term effects of CHC administration. Thus, the samples collected in each menstrual cycle were compared only to the baseline (no administration) and not to each other. Graphs were plotted in the SigmaPlot program version 12, and values were considered significantly different from each other when *p* < 0.05.

## 3. Results

No differences were found between samples collected in the luteal and follicular phases for each CHC administration. This result is not surprising since the contraceptive is expected to keep hormonal levels constant and reduce the natural cyclic variation. Thus, all further analyses were done comparing the baseline with the average of follicular and luteal samples for each CHC administration. The CHC treatment provoked physiological and hormonal alterations as well as changes in components associated with blood coagulation in the experimental subjects (*F*_1,61_ = 5.09; *p* < 0.001).

All values can be found in Table 1. Testosterone, DHT, estradiol, cortisol, lipids, fibrinogen, and hemoglobin did not differ between the baseline and treatment phases (*p* > 0.05). The constant low levels of human chorionic gonadotropin were due to the fact that the females were kept with adult males and were not pregnant. With respect to the SHBG values, the ones found in our study are lower than the values reported for another species, *Macaca fasciculans* (4.6 pg/dl) [16]. To our knowledge, no reference values for *S. libidinosus* exist in the literature.

**Figure 2:**
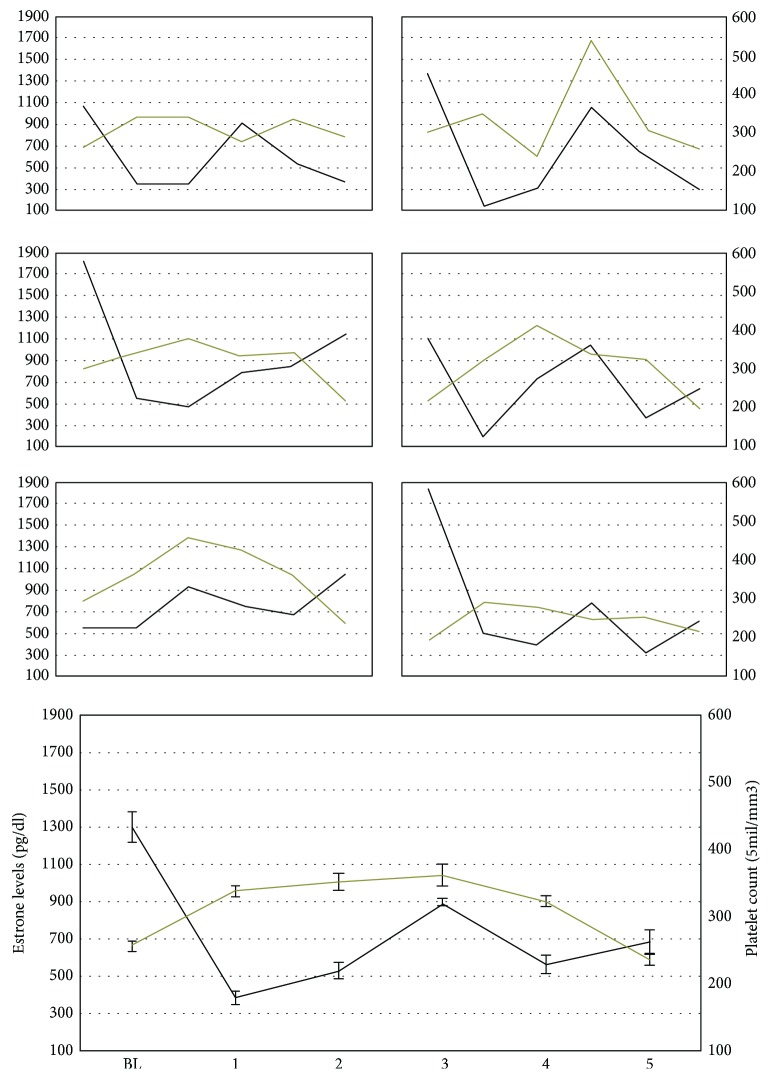
Estrone levels (black line) and platelet count (gray line) for each studied female (small graphs) and as an average of the six animals (bigger graph) in the samples collected during the baseline (BL) period and after each CHC administration. The *x*-axes of small graphs are the same as in the bigger graph. Error bars represent standard error.

Regarding estrone levels (Figure 2), a significant decrease was found when comparing BL with CHC (*F*_1,61_ = 6.95; *p* < 0.05). This result was confirmed for the five menstrual cycles when individually compared against BL (*p* < 0.002).

The number of platelets (Figure 2) was higher in the CHC phase when compared to the BL phase (*F*_1,61_ = 450.92; *p* < 0.039). This result was confirmed in the first four menstrual cycles (*p* < 0.001) but not in the last one (*p* = 0.140).

**Figure 3:**
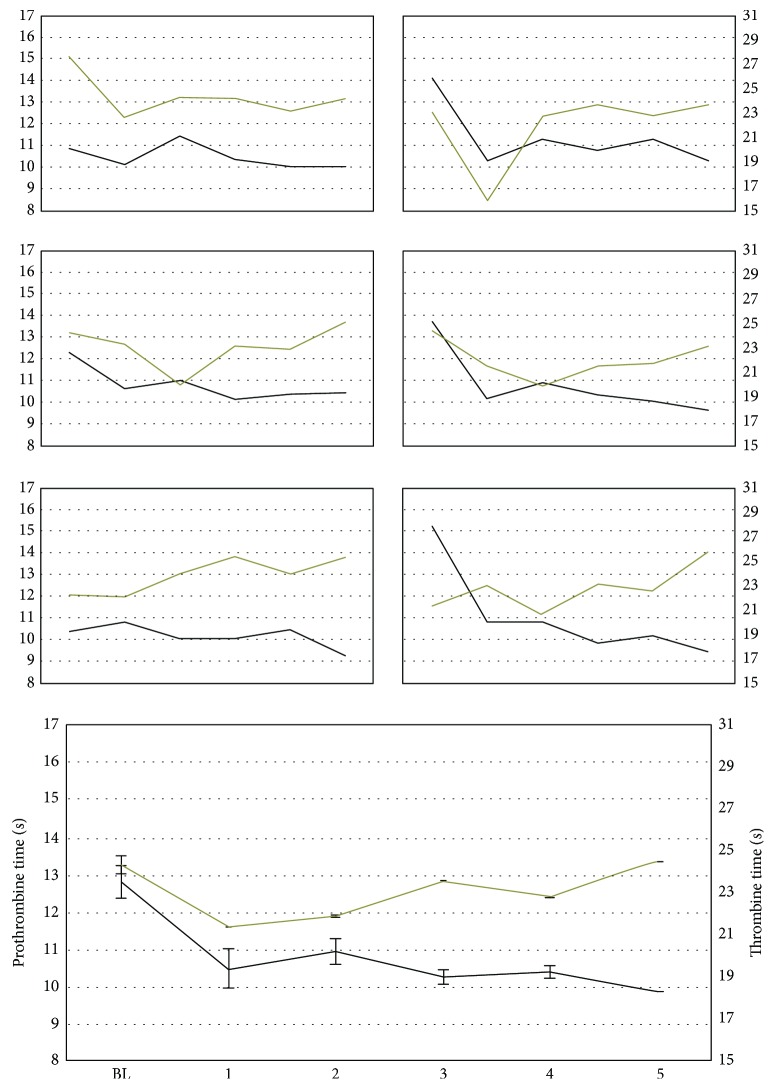
Prothrombin (black line) and thrombin (gray line) times for each studied female (small graphs) and as an average of the six animals (bigger graph) in the samples collected during the baseline (BL) period and after each CHC administration. The *x*-axes of small graphs are the same as in the bigger graph. Error bars represent standard error.

Prothrombin (Figure 3) activation time was lower in the CHC phase than in the BL phase (*F*_1,61_ = 91.77; *p* < 0.001). This finding was confirmed for all menstrual cycles when individually compared against BL (*p* < 0.001). Thromboplastin (Figure 3) activation time was also lower in the CHC phase (*F*_1,61_ = 42.94; *p* < 0.001); however, this result was only present in the first three menstrual cycles (*p* < 0.05) and not in the final two (*p* > 0.05).

## 4. Discussion

The current study aimed at investigating the physiological changes in female capuchin monkeys after treatment with a combined hormonal contraceptive. The results from blood samples collected throughout five consecutive menstrual cycles revealed that estrone levels were markedly reduced after the hormonal treatment when compared to a baseline. Although we did not directly measure FSH and LH in this study, it is possible to infer that the reduction in estrone levels was caused by the suppression of those two pituitary gonadotropins, since the use of a hormonal contraceptive reduces ovary activity.

Besides estrone, low levels of estrogen can also be associated with this suppression, due to low ovary activity during the use of hormonal contraceptive in female monkeys. Nevertheless, some of the commonly observed alterations in human females were not seen in the primates in the current study, such as a reduction in testosterone and estradiol levels [17, 18]. It should be mentioned that changes in female hormonal physiology are necessary for the prevention of follicular growth and maturation, which is the intention of the users submitted to the action of these drugs [19]. It is also emphasized that the use of hormonal contraceptives also causes a derangement in the physiology of male sex steroids, for example, a marked reduction of testosterone [17–19].

Testosterone plays an important role in maintaining female fertility because it stimulates the recruitment of follicles during their early stages [20, 21]. Experimental studies using rhesus macaques have suggested that androgens amplify the effect of FSH on folliculogenesis and have shown that the administration of testosterone in these animals increases the number of FSH receptors in the membranes of the granulosa cells, which, in turn, stimulates the initial follicular growth [22]. Moreover, during the menstrual cycle, there is an elevation of testosterone levels, which occurs in the preovulatory phase, that is, in the period before ovulation [17]. Thus, testosterone reduction may be considered to be an anovulatory effect of the contraceptives [19].

Differently, from what happens in women, no reductions in total testosterone concentrations were observed in the CHC administration phases of the current study with female monkeys. In fact, although the first contraceptive administration resulted in a numerical decline in testosterone concentration, the following CHC administration revealed a significant increase in those values. We believe that this result is due to another experiment that occurred concurrently in the Primatology Center but in another group of female prey monkeys. In this other study, doses of intranasal testosterone were administered to the monkeys. Thus, it is possible that the experimental design conducted in the other study influenced the testosterone concentrations of the monkeys that were treated with only CHC in our experiment. In this sense, it is possible that the fact that our tested females occupied the same enclosures and were physically close to each other favored the use of chemical tracks between the animals. For a more solid conclusion, the current study will need to be replicated in the absence of a concomitant testosterone study.

In women, one effect of synthetic steroids is the increase in hepatic SHBG synthesis [16, 23]. The higher the SHBG production, the lower the circulating concentration of testosterone [24]. Normally, only 0.5% of testosterone in the body has normal bioactive functions [25]. Thus, testosterone binding to proteins makes testosterone plasma concentrations low [24, 26]. However, in terms of the results obtained in the females of this study, the values of HG did not change significantly, and those of SHBG remained the same in all the experimental phases. It is possible that testosterone values have influenced the low levels of SHBG since the high testosterone levels inhibit the synthesis of SHBG [27].

In the case of estradiol, it seemed as there was a numerical reduction during the first three cycles and an increase in the last two, but these effects were not significant. The lack of statistical effects was possibly due to individual differences since the standard deviation was considerably large for estradiol levels among the studied females. It is important to note that the main objective of hormonal contraceptives is to keep the endogenous levels of estrogens and progestogens constant [28]. Usually, hormonal contraceptive users also show a marked decline in estradiol levels [20]. When women receive an intramuscular shot of CHC, a peak in estradiol level is observed lasting for up to six days. After that, the estradiol levels decline to basal levels around day 12, that is, midcycle. It is worth mentioning that, in women, there is an adaptation period where estradiol levels normalize, usually occurring from the third month of administration. These hormonal changes stop follicular growth and maturing, therefore compromising the ovulation [28].

It should be noted that, in the present study, the number of platelets remained above the baseline up to the fourth administration of HCC but reduced after the fifth one. This result is similar to that obtained in women, in whom there is a higher risk of venous thrombosis in the first three months of hormonal contraceptive use [9–12]. This risk is reduced after the fifth month of continuous use of this drug [12].

CHC administration in the current study increased the amount of platelet and decreased prothrombin and thrombin activation times. Vascular disturbances are usually associated with coagulation factors and are diagnosed by an increase in platelet count as well as changes in thrombin and prothrombin times [22]. Excess coagulation factor activation favors fibrin clot formation on vascular tissue [29], consequently leading to the development of thrombosis [22, 30]. Although it is possible that these results were affected by the testosterone experiment happening at the same time as ours, research has already shown that the use of hormonal contraceptives is associated with alterations in blood coagulation factors [8–10]. It is important to mention that the platelet number remained high until the fourth menstrual cycle of the CHC phase, but the values decreased during the fifth cycle. This result shows a strong resemblance to what has been observed for women, which have a higher risk of thrombosis during the first three months of hormonal contraceptive use [11, 31] but lower risk after the fifth month [11]. Exogenous estrogens found in combined hormonal contraceptives interact with specific endothelial cell receptors responsible for a variety of regulatory measures on the components of the vascular wall [32–35]. Some of those regulatory measures are the increase in thrombin time and amount of fibrin, the reduction of coagulation inhibitors (antithrombin, C protein, and tecidual factor inhibitor), and the reduction of plasminogen activator inhibitor. These measures can promote a complex mechanism leading to the occurrence of thrombosis [9, 36].

In face of the serious risks that hormonal contraceptives pose to the female physiology, especially with chronic use [8, 9], scientific research is seeking the technological improvement of conventional hormone methods [37, 38]. Consequently, novel methods are being developed in order to reduce the adverse and side effects, while keeping the contraceptive efficiency [38]. Some of these methods involve natural estrogen compounds (estradiol valerate) in order to reduce the risk of thrombosis [39], new hormonal formulas with different acting mechanisms [40], and a new delivery system or more specific receptor bonds that can reduce side effects caused by conventional methods [7, 40, 41]. Another new method includes the combination of an antiretroviral agent that, besides contraception, also protects the user from sexually transmitted diseases [7]. These promising methods should be carefully researched before being made available as a contraceptive choice for the users [38].

Our results show that female capuchin monkeys can be an important research model for these future studies. This is because, in addition to them already being successfully used in studies that require physiological and neuroendocrine aspects related to reproduction [14, 42], we now show that *S. libidinosus* presents the same physiological alterations as women do when administered with CHC [20].

## 5. Conclusion

In the current study, the use of CHC affected the coagulation parameters (platelet number and thrombin and prothrombin times) in female capuchin monkeys. These results are similar to what has been observed in women, therefore, reinforcing the need for new studies that seek to minimize the adverse effects of traditional hormonal contraceptive methods. However, the other physiological parameters did not change. As previously mentioned, this may have been caused because of another experiment happening at the same time that involved testosterone administration to another group of females. Despite that, the results of the current study showing significant changes in blood coagulation factors is of paramount importance, considering the aims of the pharmaceutical industry to implement innovative contraceptive methods. In this context, we conclude that female *S. libidinosus* will be crucially important as an adequate research model for these upcoming studies.

## Figures and Tables

**Table 1:** Mean ± SD of physiological variables measured during the BL (baseline) and CHC (combined hormonal contraceptive) phases in six female capuchin monkeys.

Variable	BL	Hormone Contraceptive Administration				
		1^st^ adm.	2^nd^ adm.	3^rd^ adm.	4^th^ adm.	5^th^ adm.
E2 (mg/dl)	902 ± 151	300 ± 185	457 ± 140	796 ± 400	1236 ± 1247	1641 ± 173
TT (ng/dl)	18 ± 11	10 ± 0	36 ± 47	37 ± 32	52 ± 30	26 ± 12
DHT (pg/ml)	173 ± 30	168 ± 18	193 ± 57	163 ± 35	156 ± 34	129 ± 25
Cort. (ug/dl)	142 ± 121	89 ± 41	165 ± 80	233 ± 150	259 ± 200	164 ± 151
Fibri. (mg/dl)	219 ± 17	228 ± 14	193 ± 16	225 ± 61	200 ± 14	205 ± 13
HG (mg/dl)	13 ± 1	13 ± 0.5	12 ± 0.2	12 ± 0.4	13 ± 0.2	14 ± 0.1
SHBG (Nmol/l)	2 ± 0	2 ± 0	2 ± 0	2 ± 0	2 ± 0	2 ± 0
Gona (UI/ml)	1 ± 0	1 ± 0	1 ± 0	1 ± 0	1 ± 0	1 ± 0
Lip. (mg/dl)	473 ± 27	441 ± 20	459 ± 18	532 ± 39	487 ± 46	430 ± 16

E2, estradiol; TT, testosterone; DHT, dihydrotestosterone; Cort., cortisol; Fibri., fibrinogen; HG, hemoglobin; SHBG, globulin; Gona, human chorionic gonadotropin; Lip., total lipids.
